# Microcystic Macular Edema Caused by Non-Glaucomatous Optic Atrophy: A Single-Center, Retrospective, Cohort Study in France

**DOI:** 10.3390/vision8030052

**Published:** 2024-09-06

**Authors:** Tibaut Coutureau, Jacqueline Butterworth, Damien Biotti, Pierre Fournié, Vincent Soler, Fanny Varenne

**Affiliations:** 1 Ophthalmology Department, Pierre-Paul Riquet Hospital, Toulouse University Hospital, 31059 Toulouse, France; tcoutureau@gmail.com (T.C.); butterworth.j@chu-toulouse.fr (J.B.); fournie.p@chu-toulouse.fr (P.F.); vincentsoler.oph@gmail.com (V.S.); 2 Department of Neurology, Toulouse University Hospital, 31059 Toulouse, France; biotti.d@chu-toulouse.fr; 3 Toulouse Institute for Infectious and Inflammatory Diseases, INSERM U1043, CNRS UMR 5282, 31024 Toulouse, France; 4 Faculty of Medicine, University of Toulouse III, 31400 Toulouse, France

**Keywords:** optic atrophy, microcystic macular edema, retina, fovea, inner nuclear layer, retinal nerve fiber layer, ganglion cell layer

## Abstract

Optic Atrophy (OA) can be associated with the development of microcystic macular edema (MME) in the perifoveal retinal inner nuclear layer (INL). We aimed here to retrospectively determine the prevalence of MME in patients with non-glaucomatous OA in our tertiary ophthalmology department between 2015 and 2020. We then examined how MME affected the thicknesses of the different retinal layers and the differences in demographic and clinical characteristics between those patients who developed MME and those who did not. A total of 643 eyes (429 patients) were included (mean age 45.9 ± 17.8 years, 52% female). MME developed in 95 (15%) eyes and across all etiologies of OA except for toxic/nutritional causes, but the prevalence of MME varied between the different etiologies. The development of MME was associated with thinning of the ganglion cell layer (11.0 vs. 9.6 μm; *p* = 0.001) and the retinal nerve fiber layer (10.1 vs. 9.15 μm; *p* = 0.024), with INL thickening in the 3- and 6-mm diameter areas of the central fovea. Patients developing MME had significantly worse distance best-corrected visual acuity than those not developing MME (0.62 vs. 0.38 logMAR; *p* = 0.002). Overall, the presence of MME in OA cannot be used to guide the diagnostic work-up of OA.

## 1. Introduction

Optic atrophy (OA) represents the end-stage of a wide spectrum of optic neuropathies with irreversible axonal injury arising from various hereditary or acquired etiologies. OA can develop unilaterally or bilaterally and manifest via the following ophthalmic characteristics: scotomas of variable density and size, a decline in visual acuity varying from partial to complete loss, color vision deficits, and a decrease in contrast sensitivity [1,2,3].

Spectral-domain optical coherence tomography (SD-OCT) has shown that OA is characterized by thinning of the retinal nerve fiber layer (RNFL) [4] and reduced ganglion cell layer (GCL) thickness [5,6]. Conversely, the retinal inner nuclear layer (INL) does not show thinning despite histologically proven cell depletion [7]. Nonetheless, the extremely more frequent and sometimes even systematic use of SD-OCT has made it possible to demonstrate that OA can also be associated with the presence of cavities in the INL, clinically termed microcystic macular edema (MME). MME is characterized by hyporeflective cystoid spaces located exclusively within the INL with a perifoveal distribution [8,9].

Initially discovered in patients with multiple sclerosis-associated optic neuritis [9,10], MME was later found in various causes of OA [11]: anti-aquaporin-4 antibody-positive optic neuritis [12], relapsing isolated optic neuritis [13], Leber hereditary optic neuropathy and autosomal dominant optic atrophy [14], acute anterior ischemic optic neuropathy, compressive optic neuropathy, toxic and nutritional optic neuropathy, traumatic optic neuropathy, and glaucoma [15]. The prevalence of OA-associated MME is highly study-dependent (ranging from 9% to 75% [15,16,17]), but overall the presence of MME is considered a sign of OA irrespective of its etiology [18]. So far, MME has been described as associated with a more severe stage of OA in MS [9,10], as well as with more marked thinning of the RNFL and GCL in patients with non-glaucomatous OA of variable etiology (without multiple sclerosis/neuromyelitis optica) [16]. The same study has also highlighted that patients with OA developing MME are significantly younger than those who do not develop MME [16]. Nonetheless, the underlying physiopathology of OA-associated MME remains unclear [17].

Here we report on the largest cohort of patients to date, to our knowledge, with non-glaucomatous OA with and without MME from our tertiary hospital center in France. We first aimed to determine the prevalence of MME development in OA. We also aimed to determine how the development of MME affected the thicknesses of the different retinal layers (GCL, RNFL, and INL) and examined the differences in demographic and clinical characteristics between patients developing MME and patients who did not develop MME.

## 2. Materials and Methods

### 2.1. Study Design

We retrospectively reviewed the electronic charts of all patients managed for OA in our university hospital ophthalmology department (Toulouse, France) between 1 September 2015 and 31 August 2020. To do this, we used the Ophtalmo Query search tool from the Softalmo software version 1.89.220.0 (Corilus, France) to search the key words “OA” and “papillary atrophy” in all our electronic medical records. An official waiver of ethical approval was granted from the IRB of Toulouse University Hospital given the retrospective and non-interventional nature of the study as asserted by French Jardé ethical and regulatory law. All the procedures performed were part of routine care and in accordance with institutional and national guidelines, as well as with the principles and regulations of the 1964 Declaration of Helsinki. Informed patient consent was obtained from participants and all data has been anonymized.

### 2.2. Patient Selection

The inclusion criteria were patients of any age with a confirmed diagnosis of unilateral or bilateral OA by SD-OCT detection of reduced peripapillary RNFL thickness in at least one retinal quadrant. This was with an associated asymptomatic or symptomatic functional visual loss on initial presentation to our ophthalmology department, including declines in visual acuity, and/or visual field defects, and/or color vision defects. We did not include patients with glaucoma or OA secondary to retinal damage (e.g., retinal artery occlusion, ischemic retinal vein occlusion, etc.) or retrograde trans-synaptic axonal degeneration related to brain damage. Patients with systemic or ocular pathologies that could cause cystoid maculopathies were excluded: diabetes, central retinal vein occlusion or branch retinal vein occlusion, uveitis, age-related macular degeneration (AMD), vitreoretinal interface abnormalities, hypertensive retinopathy, arterial macroaneurysm, radiation retinopathy, choroidal tumors, viral retinitis, inherited retinal dystrophy, macular telangiectasia, and Coats disease. All patients who had undergone eye surgery or been treated with drugs that could cause cystoid maculopathies (Fingolimod, prostaglandins) within the last 12 months were also excluded. Only patients who had undergone macular SD-OCT imaging combined with papillary SD-OCT imaging were included. Finally, we did not include patients for whom macular SD-OCT imaging did not allow for affirmation of the presence or absence of MME, or for whom retinal layer segmentation was difficult or of bad quality.

### 2.3. Data Collection

Patient demographic characteristics (age, sex) and medical history were collected. The severity, topography, and underlying etiology of the OA were determined. Distance best-corrected visual acuity (BCVA) was measured using the Monoyer scale and then converted into logarithm of minimum angle of resolution (logMAR) for statistical analyses. Visual acuity for counting fingers (logMAR = 1.85), hand motion (logMAR = 2.3), light perception (logMAR = 2.7), and no light perception (logMAR = 3.0) were also converted into logMAR [19]. SD-OCT detection of MME and the thicknesses of the different retinal layers (RNFL, GCL, and INL) were also collected.

### 2.4. SD-OCT and Analysis

SD-OCT imaging was performed using a combined spectral domain, high-resolution OCT with a scanning laser ophthalmoscope (SPECTRALIS^®^ HRA+OCT, Heidelberg Engineering, Heidelberg, Germany). This system allows for simultaneous infrared imaging. A scan of the optic disc was performed, including a 3.5-mm diameter ring-scan of the peripapillary RNFL to determine RNFL thickness. Then, OA was automatically detected and classed into mild, moderate, or severe according to the built-in SD-OCT device software (Heidelberg Eye Explorer version 1.12.1.0). Through the macular area, a volume scan of 25 multi cross-sections (6 × 6 mm) was performed in the horizontal direction and centered on the fovea with a slice angle of 20°. This generated an Early Treatment of Diabetic Retinopathy Study (ETDRS) macular map, which was divided into three distinct central retina areas with diameters of 1 mm, 3 mm, and 6 mm; the latter two areas were further subdivided into upper, lower, nasal, and temporal quadrants. The same built-in software allowed for automatic segmentation of the different central retinal layers. The automatic segmentation was verified by an experienced examiner (TC) and manually corrected when necessary. The diagnosis of MME was based on the identification of hyporeflective cystoid spaces exclusively within the perifoveal INL [20] on two consecutive SD-OCT sections by the same aforementioned examiner (TC).

### 2.5. Outcome Measures

We first calculated the prevalence of MME among the patients managed for OA during the study inclusion period. Then, we tested for the differences in age, sex, distance BCVA, and topography of the OA in patients who had developed MME versus patients who had not developed MME. To do this, we split the eyes of all patients into the following groups: (1) eyes with OA without MME (OA-MME group), (2) eyes with OA with MME (OA+MME group). Finally, we tested for differences in thicknesses of the central retinal layer and the different retinal layers (GCL, RNFL, and INL) between the OA-MME and OA+MME groups. We chose the central 1-mm, 3-mm, and 6-mm diameter areas to examine the central retinal and retinal INL thicknesses, but we limited examination of the central 1-mm diameter areas for the GCL and RNFL layers.

### 2.6. Statistical Analysis

Data are presented as frequencies (percentage) or means (±standard deviation). Quantitative variables were compared using the Welch’s *t*-test and qualitative variables using the Chi-squared or Fisher’s exact test when applicable. Statistical analyses were performed using GraphPad Prism version 10.2.2 Software (Boston, MA, USA). *p* values < 0.05 were considered statistically significant.

## 3. Results

### 3.1. Patient Demographic and Clinical Characteristics

A total of 1109 patients were managed for OA (all causes combined) in our tertiary hospital ophthalmology department between 1 September 2015 and 31 August 2020. Among these, 429 patients (643 eyes) were diagnosed with non-glaucomatous OA that was not secondary to retinal damage, brain damage, or systemic or ocular pathologies, and were thus included in the study. The mean age was 45.9 ± 17.8 years (6–93 years) and the sex ratio was almost equal (221 females [52%] and 208 males [48%]). The built-in SD-OCT software detected pathological RNFL thinning as moderate OA in 18% of eyes and severe OA in 81% of eyes compared to the device’s normative database. The OA topography was global in the majority (58% of eyes) and temporal in approximately a third of eyes (35%), but rarely limited to the superior (3.7% of eyes), nasal (1.2% of eyes), or inferior (3.5% of eyes) quadrants. The mean distance BCVA was 0.41 ± 0.7 logMAR and the mean RNFL thickness was 61.5 ± 15.3 μm.

### 3.2. The Prevalence of MME

**Figure 1:**
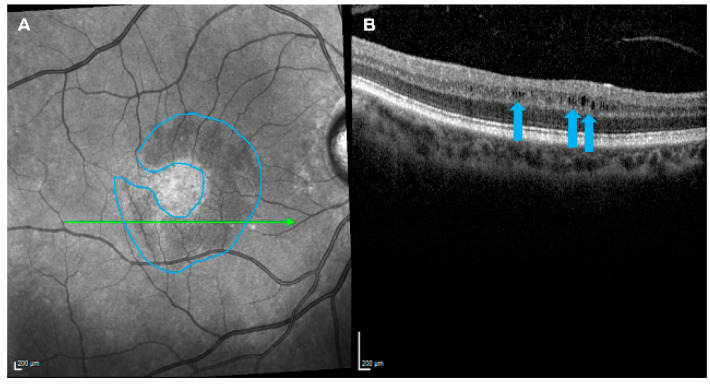
The development of microcystic macular edema in a patient with optic atrophy. A hyporeflective, perifoveal, crescent-shaped lesion (blue outline) was detected by near-infrared reflectance imaging (**A**) and found composed of hyporeflective cystoid grooves within the retinal inner nuclear layer (blue arrows) by optical coherence tomography imaging (**B**).

Among the 643 eyes from 429 patients with OA included for analysis, the majority were not associated with MME: 548 out of 643 (85%) eyes from 360 out of 429 (84%) patients; the OA-MME group. The OA was associated with MME in 95 out of 643 eyes (15%) eyes from 69 out of 429 (16%) patients; the OA+MME group. Figure 1 shows the presence of MME in a patient in the OA+MME group after identification of a hyporeflective, perifoveal crescent-shaped lesion (A) composed of hyporeflective cystoid grooves within the INL (B). The OA was bilateral in 44 out of 69 (64%) patients in the OA+MME group, among which the MME development was bilateral in 26 out of 44 (59%) patients and unilateral in 18 out of 44 (41%) patients. Among these patients with unilateral MME, the majority (*n* = 14 out of 18 [78%] patients) had developed the MME in the eye with the most severe OA (based on RNFL thickness). The MME was most frequently diffuse and localized to multiple retinal quadrants: two quadrants in 32 out of 95 (34%) eyes, three quadrants in 17 out of 95 (18%) eyes, all four quadrants in 32 out of 95 (34%) eyes, and less frequently to one quadrant in 14 out of 95 (15%) eyes.

### 3.3. Prevalence of MME According to the Etiology of the OA

Table 1 summarizes the diagnosed etiologies of OA in our patient cohort. OA was predominantly attributed to isolated inflammatory optic neuritis (16% of eyes), compressive optic neuropathy (13% of eyes), hereditary optic neuropathy (mitochondrial or nuclear DNA damage) (11% of eyes), and MS-associated inflammatory optic neuritis (11% of eyes). The following etiologies of OA were also found but at lower frequencies: secondary to intracranial hypertension (8% of eyes), acute anterior ischemic optic neuropathy (7% of eyes), traumatic optic neuropathy (6% of eyes), toxic optic neuropathy (5% of eyes), optic disc drusen (3% of eyes), myelin oligodendrocyte glycoprotein optic neuritis (2% of eyes), anti-aquaporin-4 antibody positive optic neuritis (1% of eyes), and infectious optic neuropathy (0.8% of eyes). The etiology remained undetermined for 17% of eyes.

Table 1 also highlights the varying prevalence of MME according to the etiology of the OA. The development of MME was most frequent among patients with anti-aquaporin-4 antibody positive optic neuritis (29% of eyes), with *n* = 7 out of 643 OA eyes diagnosed with this etiology, followed by patients with infectious optic neuropathy (20% of eyes), with *n* = 5 out of 643 OA eyes diagnosed with this etiology. The prevalence for the development of MME was similar for the following etiologies: acute anterior ischemic optic neuropathy (20% of eyes), traumatic optic neuropathy (20% of eyes), hereditary optic neuropathy (19% of eyes), and compressive optic neuropathy (18% of eyes). MME was less frequent among patients with isolated inflammatory optic neuritis (13% of eyes), OA secondary to intracranial hypertension (10% of eyes), MS-associated inflammatory optic neuritis (6% of eyes), optic disc drusen (6% of eyes), and myelin oligodendrocyte glycoprotein optic neuritis (6% of eyes). It is noteworthy that no patients with toxic/nutritional OA (*n* = 30) developed MME.

### 3.4. Comparisons of Demographic and Clinical Characteristics between Patients with and without MME

These results have been summarized in Table 2. We found no differences in age (44.7 ± 17.6 vs. 46.2 ± 17.8 years; *p* = 0.52) or sex (M:F sex ratios 1.4 vs. 0.88; *p* = 0.085) between patients in the OA+MME group versus the OA-MME group. Both patient groups also had similar proportions of eyes with moderate (*n* = 16 [17%] vs. *n* = 103 [19%]) and severe (*n* = 79 [83%] vs. *n* = 445 [81%]) OA (*p* = 0.12) according to the built-in SD-OCT software. Likewise, there was no difference in the topographies of the OA observed among eyes in the OA+MME group versus the OA-MME group (*p* = 0.52). Briefly, the majority of eyes had developed diffuse OA in both the OA+MME group (*n* = 53 [56%]) and the OA-MME group (*n* = 318 [58%]), followed by temporal OA (OA+MME group: *n* = 39 [41%] eyes; OA-MME group: *n* = 185 [34%] eyes). Superior, nasal, and inferior topographies were rarer (≤4% of eyes) for eyes in both groups. Mean RNFL was significantly thinner among eyes in the OA+MME group than eyes in the OA-MME group (55.9 ± 16.4 vs. 62.5 ± 14.9 μm; *p* < 0.0001) and mean distance BCVA was significantly worse among eyes in the OA+MME group than eyes in the OA-MME group (0.62 ± 0.8 vs. 0.38 ± 0.7 logMAR; *p* = 0.0021).

### 3.5. Retinal Layer Analysis

Comparisons of the different retinal layer thicknesses between patients with and without MME are found in Table 3. The mean central retinal thickness was similar in patients in the OA+MME and OA-MME groups for both the central 1-mm (263 ± 30.4 vs. 263 ± 20.9 μm; *p* = 0.79) and 3-mm (312 ± 17.6 vs. 309 ± 21.6 μm; *p* = 0.09) diameter areas. However, the mean central retinal thickness was significantly thicker in the 6-mm diameter area for patients in the OA+MME group (277 ± 11.6 vs. 273 ± 15.8 μm; *p* = 0.001). Regarding the layers of the central retina, the mean retinal INL thickness was similar in patients in the OA+MME and OA-MME groups (20.9 ± 8.4 vs. 19.2 ± 5.9 μm; *p* = 0.058) for the 1-mm diameter area. On the other hand, the mean INL was significantly thicker for patients in the OA+MME group for both the 3-mm (49.9 ± 6.5 vs. 42 ± 4.3 μm; *p* = 0.0001) and 6-mm (38.4 ± 4.0 vs. 33.9 ± 3.4 μm; *p* = 0.0001) diameter areas. The GCL (9.6 ± 3.4 vs. 11 ± 4.2 μm; *p* = 0.001) and RNFL (9.2 ± 3.9 vs. 10.1 ± 3.2 μm; *p* = 0.024) were significantly thinner for patients in the OA+MME group than in the OA-MME group for the 1-mm diameter area.

## 4. Discussion

We report here, to our knowledge, on the largest cohort of patients to date managed for non-glaucomatous OA with and without MME in our tertiary hospital center in France. Overall, we found no difference in age or sex between patients with OA who developed MME compared to patients with OA who did not develop MME. Likewise, OA severity subgrouping and topography were similar among patients developing or not developing MME. On the other hand, patients with OA who had developed MME had significantly worse visual acuity, their RNFL and GCL were significantly thinner, and the INL was significantly thicker given the presence of the cystoid cavities. Hence, the MME had developed in patients with more severe OA. Finally, MME developed across all etiologies of OA except for toxic/nutritional causes, but the prevalence of MME varied greatly between the different etiologies.

Our study found a prevalence of 15% for MME development among eyes with non-glaucomatous OA. This prevalence is higher than the prevalence found by Abegg et al. [16] in 2014 (8.8% of eyes among 16 eyes with OA), but lower than the prevalence found by Pott et al. [17] in 2016 (18.9% of eyes among 90 eyes with OA) and by Wolff et al. [15] in 2014 (35.3% of eyes among 71 eyes with OA). These differences in prevalence could at least partly be explained by the small patient numbers of the aforementioned studies compared to our larger cohort of patients with OA here. Inter-study variations in the prevalence of MME in OA could also be affected by differences in the timing of diagnosis and follow-up examinations of MME due to differences in study design. Notably, Pott et al. [17] and Wolff et al. [15] performed prospective patient inclusion based on patients with a known history of OA and known defects in their RNFL.

MME developed across all etiologies of OA in our study except for toxic/nutritional causes, but the prevalence of MME varied between the different etiologies (refer to Table 1). Despite high inter-study differences in the prevalence of MME in OA, a trend for low prevalence of INL changes can be observed in some etiologies of OA [15,16,17]. In this light, we found MME in only 6% of eyes with MS-related OA. This finding is similar to the prevalence of 2.8% of eyes described in the literature review by Kessel et al. [21]. Conversely, a high proportion of eyes (29%) developed MME among patients with anti-aquaporin-4 antibody-positive optic neuritis, but the prevalence of this etiology of OA was very low in our cohort (*n* = 7 out of 643 [1%] eyes). This finding requires validation, for instance, in a multi-centric French study. Hereditary OA with MME developed in 19% of eyes in our study compared to 65.4% reported by Kessel et al. [21]. Finally, we classed a high proportion of patients (*n* = 108 out of [643] 17% eyes) as having an undetermined etiology of OA, of whom 20% developed MME. We can thus conclude from this finding that the presence of MME cannot guide the diagnosis of a specific etiology of OA [18] given this high proportion of undetermined OA and the fact that MME was found among all OA etiologies, except toxic/nutritional OA. Importantly, this means, during the substantial diagnostic work-up, that the detection or development of MME cannot be used to eliminate any etiologies of OA, or even allow us to suspect one etiology over others.

In terms of retinal layer thicknesses, the mean RNFL thickness was 61.5 ± 15.3 μm for our entire cohort of patients with OA, thus approximately 35 μm thinner than healthy eyes from white adults in Germany, for whom a mean RNFL thickness of 97.2 ± 9.7 μm has been reported, measured using the same device [22]. In the present study, we found that the development of MME was associated with thinning of both the GCL (mean reduction of −1.4 μm) and RNFL (mean reduction of −0.9 μm) for the 1-mm diameter area (foveolar region), hence in eyes with more severe OA [5,6,23]. Conversely, the INL was significantly thicker in the OA+MME group than in the OA-MME for the 3-mm and 6-mm diameter areas as described in the literature [14], but not in the foveolar region as expected. Indeed, MME in OA extends 500–2300 µm from the foveal center [8,9,15,24]. We also found that patients with OA+MME had significantly poorer visual acuity than patients with OA-MME. However, it has already been reported that visual acuity is not related to the development of MME, but rather to the severity of the OA. Indeed, regression of MME following treatment with Diamox does not result in any improvement in visual acuity [25] and edema has shown to develop in the more affected eye among the majority of patients with bilateral OA [15,23]. Furthermore, MME has not been found in healthy eyes [21], reinforcing the hypothesis that OA is necessary for the development of MME [24]. Finally, we did not find any significant differences in age [17] or sex between patients who developed or who did not develop MME.

There are currently several hypotheses for the pathophysiological mechanisms underlying MME development. Firstly, an inflammatory origin appears unlikely as MME is found across all etiologies of OA, and the onset of MME can often be late in relation to the onset of the OA. Moreover, angiography, when performed, has not revealed macular diffusion [8,14,15,26] and so MME could indeed be more accurately classified as a non-vasogenic cystoid maculopathy [27]. Another hypothesis describes how vitreo-macular traction is partly responsible for schisis localized solely to the INL. Indeed, cystoid maculopathies confined to the INL in patients with epiretinal membrane or vitreo-macular traction can develop independently of OA [14,28,29]. Reduced GCL and RNFL thicknesses, combined with cell loss within the INL and vitreo-macular attachment, could be the underlying cause. Whether this mechanical contribution is related or not to retrograde degeneration of bipolar cells via a trans-synaptic pathway, as a result of ganglion cell loss, still remains under debate [18] but is supported by early findings of cystoid cavities within the INL in primates with damage to the optic nerve or optic chiasm [30,31]. The diffuse topographical distribution of retinal nerve fiber, combined with MME-localized thinning of the GCL and RNFL, are also arguments favoring this hypothesis [14]. Accordingly, similar to non-exudative AMD, which can be complicated by intra-retinal cystoid spaces in a context of retinal cell loss, trans-synaptic retrograde degeneration could explain the development of “empty” micro-cystoid spaces in OA [27]. It has also been suggested that Müller cells could be the cause. Müller cell bodies are located in the INL and are involved in the absorption of intraretinal fluids; cystoid cavities would therefore not be due to a rupture in the blood–retinal barrier, but to a reduction in fluid resorption due to Müller cell dysfunction [32]. In addition, Müller cells express aquaporins [32]. The high incidence of MME in neuromyelitis optica could therefore be related to the presence of anti-AQP4 antibodies.

One study limitation is the retrospective design and consequently a low frequency of follow-up consultations. In addition, macular SD-OCT is not systematically performed during every follow-up of OA in our center. Indeed, follow-up data were only collected for 45% of eyes in the OA+MME group (*n* = 43/95). This meant that we could not accurately determine the time between the onset of OA and the development of MME. In addition, among patients for whom we collected follow-up data, the follow-up delays were highly variable, ranging from 2 months to several years (40.2 ± 95 months). To date, Abegg et al. [16] have found that MME remained stable in 7 out of 9 patients (5.3 ± 8 months follow-up), but Saidha et al. [10] have highlighted fluctuations in 5 out of 12 patients with MS-related OA, with MME worsening or improving in the remaining eyes (25.8 ± 9.1 months follow-up). Large prospective studies are therefore required to accurately investigate both the onset of MME in OA and the evolution of MME over time. Another study limitation lies in the fact that Toulouse University Hospital has an emergency ophthalmology department, and so we can expect a high proportion of patients presenting with OA to have been diagnosed in the early phase of OA development. This means not all patients with OA developing MME can be accounted for in our cohort.

## 5. Conclusions

MME was found in 16% of patients with non-glaucomatous OA. Development of MME was associated with thinning of the GCL and the RNFL, and caused INL thickening in the central 3-mm and 6-mm retinal areas, thus confirming the perifoveal distribution of MME. Patients developing MME had significantly worse visual acuity than those not developing MME. MME developed across all etiologies of OA except for toxic/nutritional causes, but the prevalence of MME varied greatly between the different etiologies. We therefore suggest that the detection or development of MME cannot be used to guide the diagnosis of a specific etiology of OA during diagnostic work-up in the clinic.

## Figures and Tables

**Table 1:** Prevalence of microcystic macular edema according to the etiology of the optic atrophy.

Number of Eyes, *n* (%)	With OA 643 (100%)	With OA+MME 95 (15%)
Etiology of optic atrophy, *n* (%) eyes per etiology with OA		
Inflammatory	197 (31%)	21 (22%)
MS-associated inflammatory optic neuritis	69 (11%)	4 (6%)
Myelin oligodendrocyte glycoprotein optic neuritis	16 (2%)	1 (6%)
Anti-aquaporin-4 antibody-positive optic neuritis	7 (1%)	2 (29%)
Isolated inflammatory optic neuritis	100 (16%)	13 (13%)
Infectious optic neuropathy	5 (0.8%)	1 (20%)
Hereditary	69 (11%)	13 (19%)
Secondary to intracranial hypertension	49 (8%)	5 (10%)
Compressive	87 (13%)	16 (18%)
Optic disc drusen	18 (3%)	1 (6%)
Ischemic	44 (7%)	9 (20%)
Traumatic	41 (6%)	8 (20%)
Toxic and nutritional	30 (5%)	0 (0%)
Undetermined	108 (17%)	22 (20%)

MME: microcystic macular edema; MS: multiple sclerosis; OA: optic atrophy.

**Table 2:** Comparisons of demographic and clinical characteristics between patients diagnosed with optic atrophy who developed and who did not develop microcystic macular edema.

	OA+MME	OA-MME	*p* Value
Number of patients, *n* (%)	69 (16%)	360 (84%)	
Mean age, years (SD)	44.7 (17.6)	46.2 (17.8)	0.52 ^a^
Sex, *n* (% per group)			0.085 ^c^
Female	29 (42%)	192 (53%)	
Male	40 (58%)	168 (47%)	
Number of eyes, *n* (%)	95 (15%)	548 (85%)	
Severity of OA, *n* (% per group)			0.12 ^b^
Moderate	16 (17%)	103 (19%)	
Severe	79 (83%)	445 (81%)	
OA topography, *n* (% per group)			0.52 ^b^
Diffuse	53 (56%)	318 (58%)	
Temporal	39 (41%)	185 (34%)	
Superior	2 (2.1%)	22 (4%)	
Nasal	0 (0%)	8 (1.5%)	
Inferior	1 (1.1%)	15 (2.7%)	
Mean distance BCVA, logMAR (SD)	0.62 (0.8)	0.38 (0.7)	0.002 ^a^
Mean RNFL thickness, μm (SD)	55.9 (16.4)	62.5 (14.9)	0.0001 ^a^

BCVA: best-corrected visual acuity; logMAR: logarithm of minimum angle of resolution; MME: microcystic macular edema; OA: optic atrophy; RNFL: retinal nerve fiber layer; SD: standard deviation; ^a^: Welch’s *t*-test; ^b^: Fisher’s exact test; ^c^: Chi-squared test.

**Table 3:** Comparisons of the thicknesses of the central retinal layer and the different retinal layers between patients with optic atrophy who developed and who did not develop microcystic macular edema.

	OA+MME *n* = 95 (15%) Eyes	OA-MME *n* = 548 (85%) Eyes	*p* Value ^a^
Mean central retinal thickness, μm (SD)			
1-mm diameter area	263 (30.4)	263 (20.9)	0.79
3-mm diameter area	312 (17.0)	309 (21.6)	0.09
6-mm diameter area	277 (11.6)	272 (15.8)	0.001
Mean retinal inner nuclear layer thickness, μm (SD)			
1-mm diameter area	20.9 (8.4)	19.2 (5.9)	0.058
3-mm diameter area	49.9 (6.5)	40.9 (4.3)	0.0001
6-mm diameter area	38.4 (4.0)	33.2 (3.4)	0.0001
Mean ganglion cell layer thickness, μm (SD)			
1-mm diameter area	9.6 (3.4)	11.0 (4.2)	0.001
Mean retinal nerve fiber layer thickness, μm (SD)			
1-mm diameter area	9.2 (3.9)	10.1 (3.21)	0.024

MME: microcystic macular edema; OA: optic atrophy; SD: standard deviation; ^a^: Welch’s *t*-test.

## Data Availability

All datasets generated and analyzed are available on reasonable request from the corresponding author.
